# Constrictive pericarditis in cystic fibrosis: a case report of an emerging cardiovascular complication successfully managed with off-pump pericardiectomy

**DOI:** 10.1093/ehjcr/ytag734

**Published:** 2026-10-06

**Authors:** Defne Elif Alev, Andrea D’Alessio, Rana Ahmed Sayeed

**Affiliations:** Department of Professional and Continuing Education, University of Cambridge, Madingley Hall, Madingley, Cambridge CB3 8AQ, UK; Department of Cardiothoracic Surgery, Oxford University Hospitals, John Radcliffe Hospital, Headley Way, Headington, Oxford OX3 9DU, UK; Department of Cardiothoracic Surgery, Oxford University Hospitals, John Radcliffe Hospital, Headley Way, Headington, Oxford OX3 9DU, UK; Department of Cardiothoracic Surgery, Oxford University Hospitals, John Radcliffe Hospital, Headley Way, Headington, Oxford OX3 9DU, UK

**Keywords:** Constrictive pericarditis, Cystic fibrosis, Pericardiectomy, Off-pump cardiac surgery, Multidisciplinary care, Case report

## Abstract

**Background:**

Constrictive pericarditis (CP) is rarely reported in people with cystic fibrosis (CF) and may be overlooked because dyspnoea, fatigue, oedema, and reduced exercise tolerance overlap with pulmonary exacerbation. As survival improves with highly effective CF transmembrane conductance regulator modulator therapy, cardiovascular causes of cardiorespiratory symptoms may become increasingly relevant in adults with CF. This case highlights the importance of recognizing CP in this population and individualizing surgical decision-making.

**Case summary:**

A 33-year-old woman with homozygous F508del CF, stable on elexacaftor/tezacaftor/ivacaftor, presented with chest pain, left scapular discomfort, breathlessness, night sweats, and signs of systemic venous congestion. Computed tomography pulmonary angiography excluded pulmonary embolism but demonstrated pericardial calcification, thickening, bilateral pleural effusions, and a small pericardial effusion. Transthoracic echocardiography, cardiac magnetic resonance imaging, and invasive haemodynamic assessment confirmed chronic CP. Following treatment of an acute infective/inflammatory episode and recovery from congestive hepatopathy, she underwent off-pump complete pericardiectomy via median sternotomy with phrenic-to-phrenic resection. She was extubated within 1 h, received immediate chest physiotherapy, and was discharged on postoperative day 4. A reactive pleural effusion on day 28 resolved with drainage and antibiotics. At 4 months, oedema had resolved, exercise tolerance had improved, quality-of-life scores improved, and echocardiography showed no recurrent constriction.

**Discussion:**

CP should be considered in people with CF when symptoms are disproportionate to pulmonary disease severity. CF should not automatically preclude pericardiectomy; in selected patients, CF-specific perioperative planning may enable definitive surgery with clinical and quality-of-life benefit.

Learning points

- Pericardial calcification is present in around one in five adults with cystic fibrosis (CF); disproportionate breathlessness or peripheral oedema should prompt investigation for constrictive pericarditis (CP).
- Multidisciplinary assessment and individualized care are important in patients with the rare association of CF and CP.
- CF alone should not preclude pericardiectomy; in appropriately selected patients, definitive surgical treatment may improve symptoms and quality of life.

## Introduction

Survival in cystic fibrosis (CF) has improved substantially since the introduction of highly effective CFTR modulator therapy, with median predicted survival now projected to reach 65 years for those born between 2020 and 2024.^1^ As people with CF live longer, previously rare extrapulmonary complications are increasingly encountered.^2,3^ Constrictive pericarditis (CP), in which a fibrotic, non-compliant pericardium impairs ventricular filling, is seldom described in CF,^4–7^ and complete pericardiectomy is exceptionally rare.^6,7^ Diagnosis may be delayed because dyspnoea, fatigue, peripheral oedema, and reduced exercise tolerance overlap with pulmonary exacerbation.

We report chronic CP in an adult with CF treated by off-pump complete pericardiectomy and suggest that CF should not of itself exclude patients from definitive surgical treatment.

## Summary figure

**Figure:**
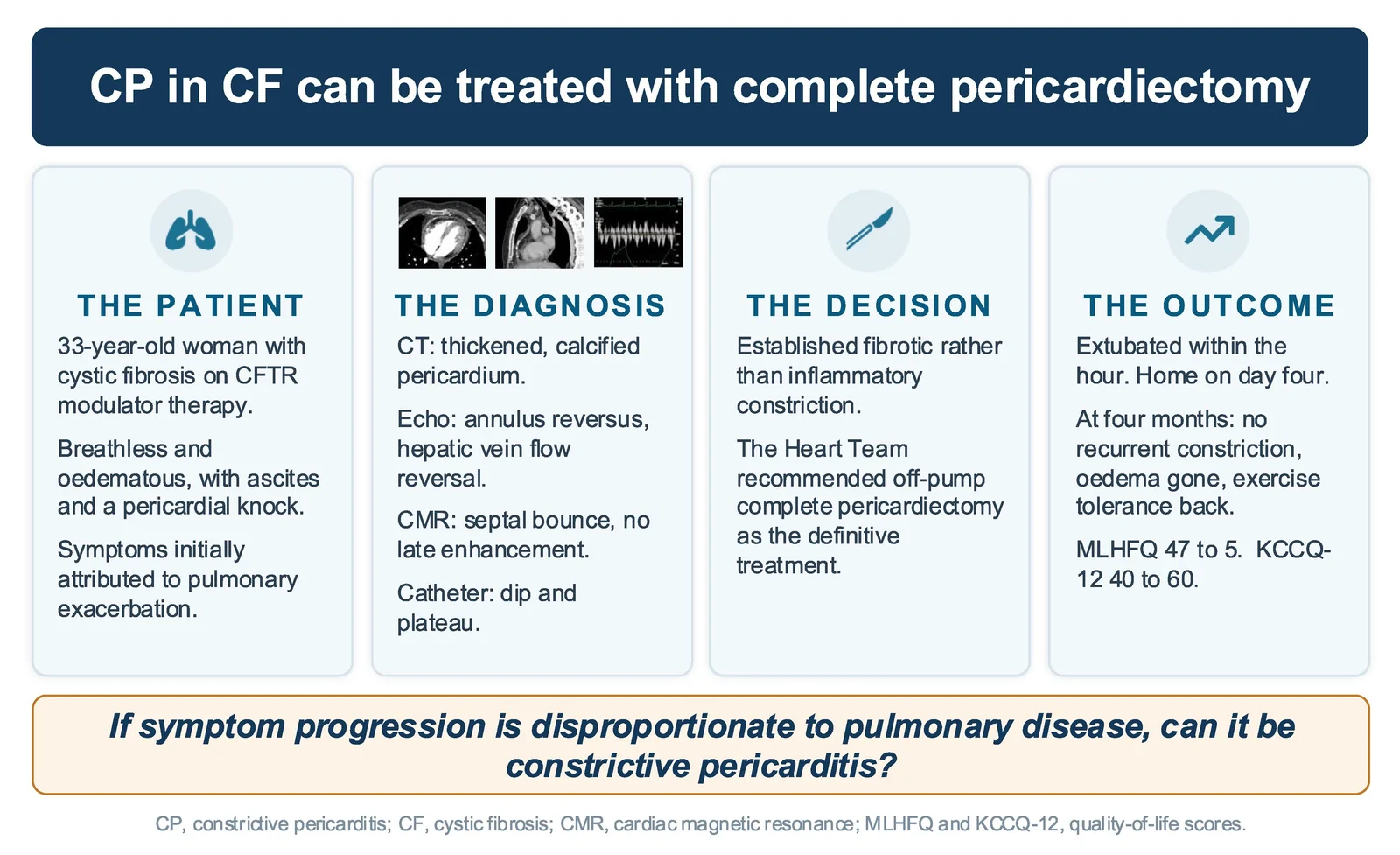


## Case presentation

A 33-year-old woman with homozygous F508del CF presented with several days of chest and left scapular pain, breathlessness, and night sweats. Lung function was stable on elexacaftor/tezacaftor/ivacaftor; comorbidities included pancreatic insufficiency, CF-related diabetes, and chronic *Pseudomonas aeruginosa* and *Staphylococcus aureus* colonization. CT pulmonary angiography excluded pulmonary embolism but showed bilateral pleural effusions, a small pericardial effusion, and pericardial thickening with calcification. (*Figure 1*)

**Figure 1:**
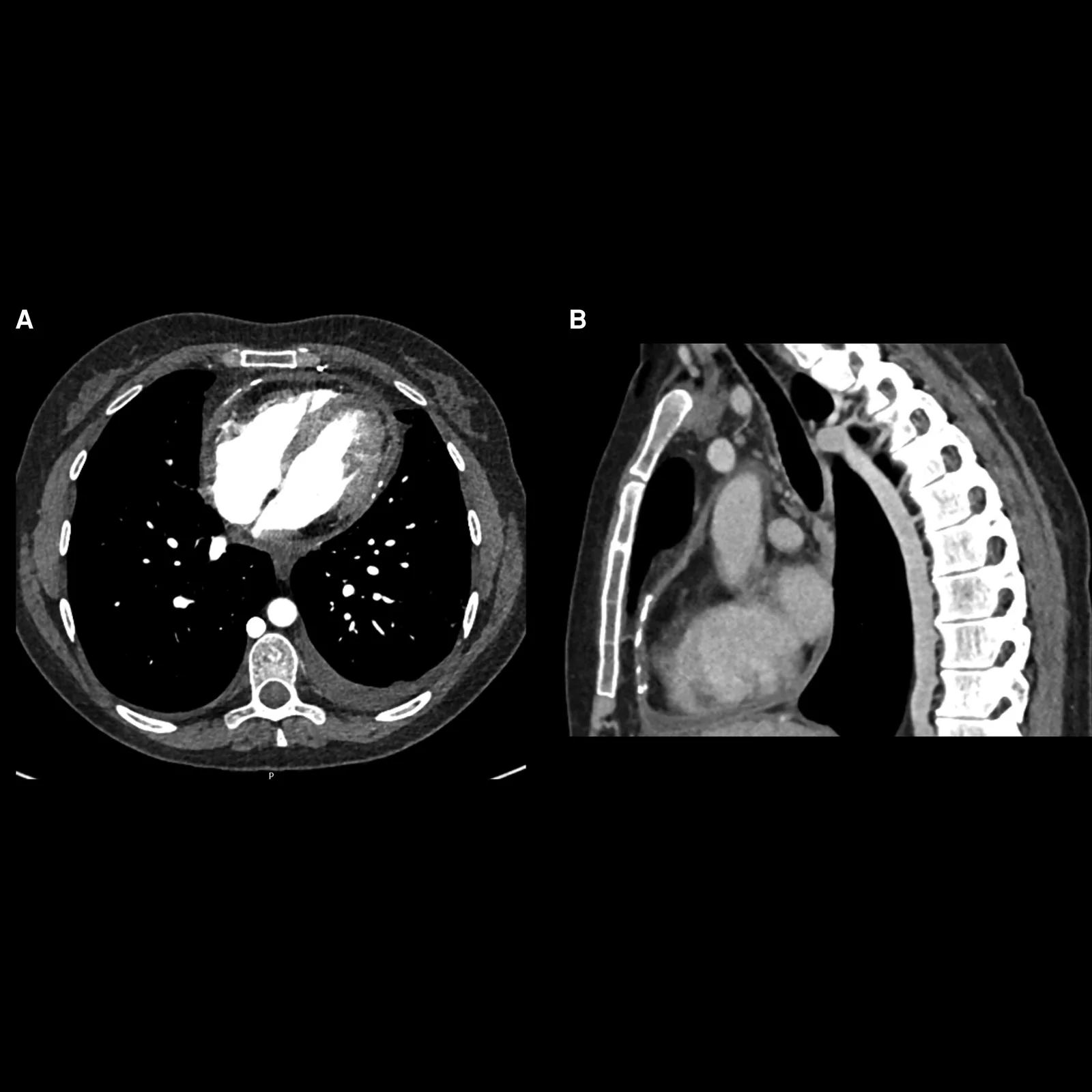
Contrast-enhanced CT, axial (*A*) and sagittal (*B*): pericardial thickening and calcification.

She had elevated jugular venous pressure, hepatomegaly, ascites, bilateral lower-limb oedema, and a pericardial knock. C-reactive protein was 290 mg/L and B-type natriuretic peptide 1130 pg/mL, with congestive hepatopathy that improved with volume optimization.

Serial echocardiography demonstrated persistent constrictive physiology: inspiratory septal shift; expiratory hepatic vein diastolic flow reversal (*Figure 2*); medial early diastolic mitral annular velocity exceeding lateral (medial e′ 18.0 cm/s, lateral e′ 14.0 cm/s), consistent with annulus reversus (*Figure 3*); equal systolic and diastolic pulmonary venous flow; and a non-dilated but non-collapsing inferior vena cava.

**Figure 2:**
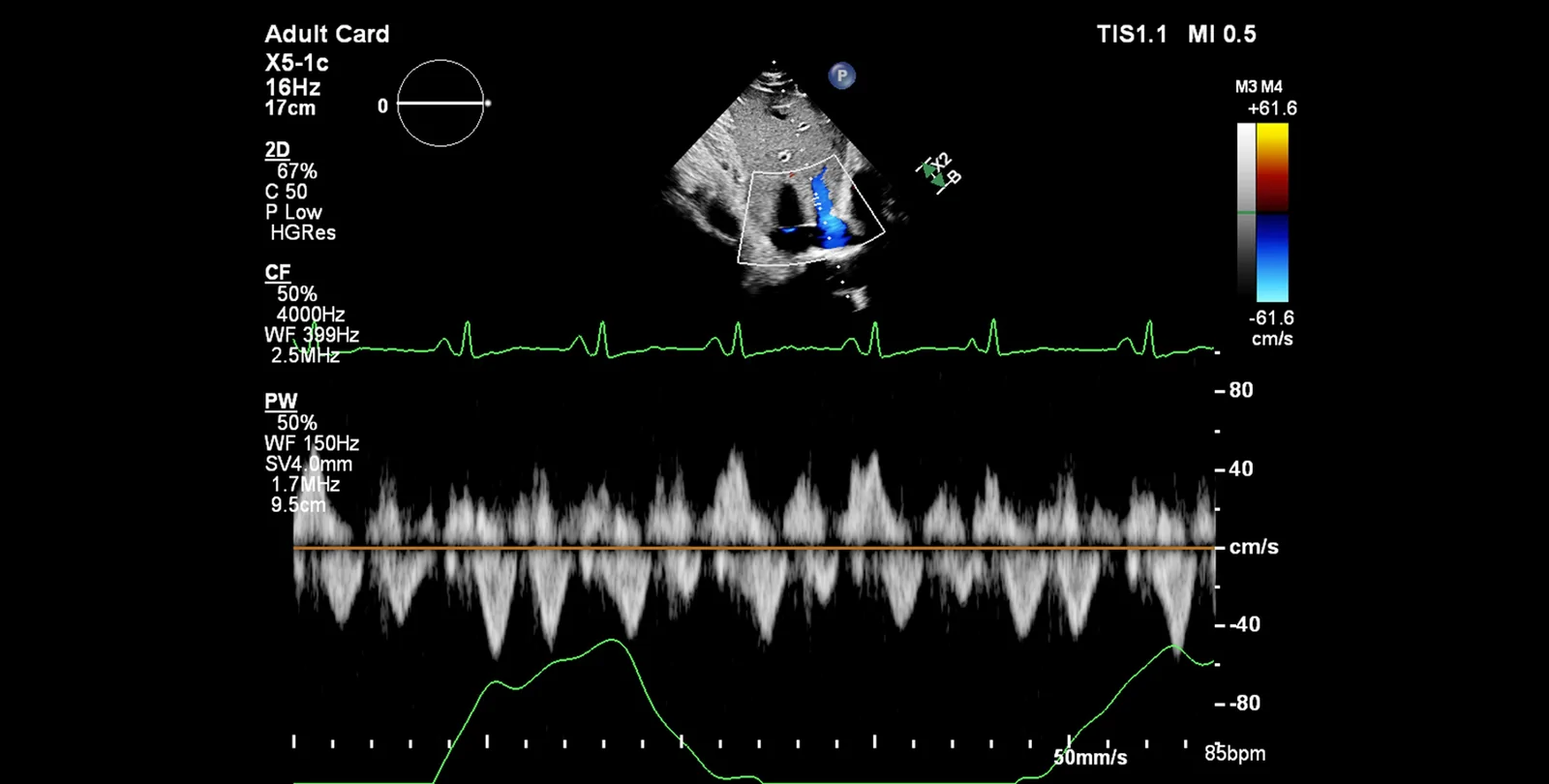
Hepatic vein Doppler: expiratory diastolic flow reversal.

**Figure 3:**
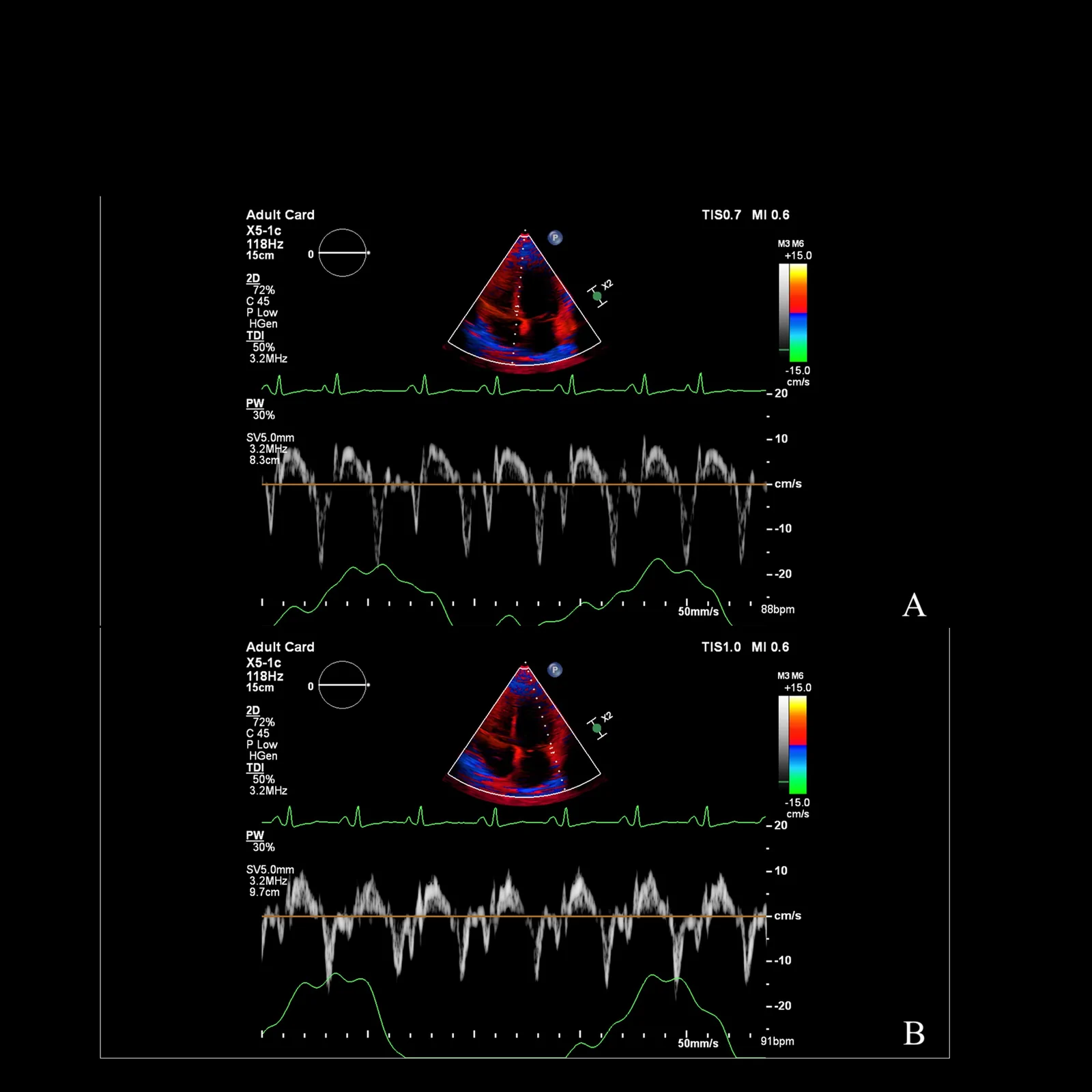
Tissue Doppler at the medial (*A*) and lateral (*B*) mitral annulus: medial e′ exceeds lateral e′, consistent with annulus reversus.

Cardiac magnetic resonance demonstrated prominent visceral and parietal pericardial thickening, inspiratory diastolic septal flattening indicating ventricular interdependence, and a septal bounce. Biventricular volumes and function were low-normal (LVEF 56%, RVEF 62%), without myocardial or pericardial late gadolinium enhancement. Cardiac catheterization demonstrated elevated ventricular diastolic pressures with a dip-and-plateau waveform.

The presentation suggested an infective or inflammatory episode superimposed on chronic CP; improvement in inflammatory markers with antimicrobials supported an acute systemic inflammatory component. Differential causes included tuberculous or other infection, connective tissue disease, and chronic inflammation related to recurrent pulmonary infection. However, investigation did not identify a specific aetiology. Tests for tuberculosis (interferon-gamma release assay) and *Aspergillus*, and a full autoimmune antibody panel, were all negative. Her medications contained no agent associated with pericardial constriction,^8^ and CFTR modulator therapy has not been associated with pericardial calcification.^9^

At Heart Team meeting, pericardiocentesis was judged unsuitable: the effusion was small, hepatomegaly limited a safe subcostal approach, and the physiology was constrictive rather than tamponade. Established calcification without pericardial enhancement indicated fibrotic rather than actively inflammatory constriction, unlikely to respond to anti-inflammatory therapy. Given the chronicity and her young age, pericardiectomy was recommended as the most definitive and durable option. Surgery was planned electively once systemic inflammation, pulmonary status, and hepatic congestion had stabilized. She was managed with low-dose furosemide, pleural drainage, and treatment of intercurrent infection, with symptoms and constrictive physiology persisting throughout.

Preoperative FEV_1_ was 1.11 L (42% predicted) and FVC 2.41 L (79% predicted). She underwent complete pericardiectomy via median sternotomy without cardiopulmonary bypass, phrenic-to-phrenic from diaphragm to great vessels. The pericardium was thickened, fibrotic, calcified, and moderately adherent to the epicardium. Histopathology showed hypocellular fibrosis with foci of coarse calcification and patchy mild chronic inflammation, no granulomas were seen, and culture of resected tissue yielded no growth.

She was extubated within 1 h, with immediate chest physiotherapy, early mobilization, and continuation of CF-specific respiratory care, and discharged on day 4. A reactive left pleural effusion on day 28 resolved with ultrasound-guided drainage and antibiotics. At 4 months, peripheral oedema had resolved, exercise tolerance had improved, and echocardiography showed no recurrent constriction. Minnesota Living with Heart Failure Questionnaire score improved from 47 to 5 and Kansas City Cardiomyopathy Questionnaire-12 score from 40 to 60.

## Discussion

Clinically overt CP in CF appears exceptionally rare, with the published literature limited to isolated case reports.^4–7^ It may nevertheless be under-recognized because its manifestations overlap with those of pulmonary exacerbations, potentially delaying diagnosis.^3,7^ Subclinical pericardial involvement, however, is considerably more common: a recent cohort identified pericardial calcification on chest computed tomography in 18% of adults with CF, compared with 1% of matched controls, 2% of patients with previous tuberculosis, and 4% of those with primary ciliary dyskinesia (*P* < 0.001). Although the underlying mechanism remains unknown, these comparisons make chronic pulmonary infection adjacent to the pericardium a less likely explanation and raise the possibility of a CF-specific process.^9^ Our patient may therefore represent the severe end of a predominantly subclinical spectrum that could become increasingly apparent as the CF population ages. Systemic venous congestion or dyspnoea disproportionate to pulmonary disease should prompt careful clinical assessment, multimodality imaging, and multidisciplinary management.^4–7^

Complete pericardiectomy has rarely been reported in CF.^6,7^ Published management has been predominantly medical or drainage based.^4,5^ Complete pericardiectomy is a high-risk operation,^10^ and its rarity in CF together with the minimal published experience may understandably deter referral for surgery. Median sternotomy impairs cough, and cardiac surgery risks atelectasis, pneumonia, and pleural effusion, any of which may precipitate exacerbation;^11^ our patient required drainage and antibiotics for a reactive effusion on day 28. Her recovery likely reflects stable preoperative lung function and CF-tailored planning: avoidance of cardiopulmonary bypass and its inflammatory and haemodilutional sequelae,^12^ complete resection, extubation within 1 h, and immediate, uninterrupted airway clearance.^13^

The 2025 ESC Guidelines list life-limiting disease among potential contraindications to pericardiectomy;^14^ that caveat should be applied to the individual rather than to the diagnosis. In carefully selected adults with CF and stable pulmonary function, complete pericardiectomy can provide substantial improvements in symptoms, functional capacity, and quality of life when delivered through a multidisciplinary Heart Team involving respiratory medicine, anaesthesia, and CF physiotherapy.

Limitations include the single-case design and the inability to establish the aetiology definitively. Follow-up extends to 4 months, which we consider sufficient to capture recovery from sternotomy and the haemodynamic benefit of surgery; beyond this, change in functional status is more likely to reflect progression of the underlying CF, which pericardiectomy cannot modify.

## Patient perspective

Immediately after surgery, disruption of my usual cystic fibrosis routine affected how I felt and scored my quality of life. After returning home and restarting physiotherapy, my breathing, swelling, and exercise tolerance clearly improved. I am very grateful to have had the operation.

## Data Availability

The data underlying this article cannot be shared publicly due to the privacy of the individual described in this case report. The data will be shared on reasonable request to the corresponding author.

## Lead author biography

Defne Elif Alev graduated with an MD from Università Cattolica del Sacro Cuore, Rome, where her dissertation examined outcomes following minimally invasive aortic valve replacement in patients with obesity. She undertook Foundation Training in the Thames Valley Deanery, during which she encountered this patient while working in cardiothoracic surgery at John Radcliffe Hospital, Oxford. She is currently a Clinical Teaching Fellow at Royal Papworth Hospital, Cambridge, leading ultrasound and simulation-based medical education alongside clinical work in cardiothoracic surgery. Her interests include aortic surgery and interprofessional surgical education. She is a member of the Society for Cardiothoracic Surgery(SCTS).

## Notes

**Consent:** Written informed consent was obtained from the patient for publication of this case report, including all clinical data and quality-of-life outcomes. Formal research ethics committee approval was not required for this single-patient case report. Written informed consent for publication was obtained from the patient.
